# A Three-Site Clinical Feasibility Study of a Flexible Functional Electrical Stimulation System to Support Functional Task Practice for Upper Limb Recovery in People With Stroke

**DOI:** 10.3389/fneur.2019.00227

**Published:** 2019-03-20

**Authors:** Christine Smith, Mingxu Sun, Laurence Kenney, David Howard, Helen Luckie, Karen Waring, Paul Taylor, Earl Merson, Stacey Finn, Sarah Cotterill

**Affiliations:** ^1^Department of Allied Health Professions, Sheffield Hallam University, Sheffield, United Kingdom; ^2^Centre for Health Sciences Research, University of Salford, Salford, United Kingdom; ^3^School of Electrical Engineering, University of Jinan, Jinan, China; ^4^School of Computing, Science and Engineering, University of Salford, Salford, United Kingdom; ^5^The National Clinical FES Centre, Salisbury District Hospital, Salisbury, United Kingdom; ^6^Centre for Biostatistics, School of Health Sciences, Faculty of Biology, Medicine and Health, The University of Manchester, Manchester, United Kingdom

**Keywords:** functional electrical stimulation, upper limb, stroke, rehabilitation, clinical investigation, usability, functional activity

## Abstract

**Introduction:** Of those people who survive a stroke, only between 40 and 70% regain upper limb dexterity. A number of reviews have suggested that functional electrical stimulation (FES) may have a beneficial effect on upper limb motor recovery. In light of the promise offered by FES and the limitations with current systems a new system was developed (FES-UPP) to support people with stroke (PwS) to practice a range of voluntary controlled, FES-assisted functional activities.

**Objective:** This paper reports on a three center clinical investigation with the primary aim of demonstrating compliance of the new FES system with relevant essential requirements of the EU Medical Device Directive, namely to evaluate whether use of the FES-UPP enables PwS to perform a wider range of functional activities, and/or perform the same activities in an improved way.

**Design:** Clinical investigation and feasibility study.

**Settings:** An in-patient stroke unit, a combined Early Supported Discharge (ESD) and community service, and an outpatient clinic and in-patient stroke unit.

**Participants:** Nine therapists and 22 PwS with an impaired upper limb.

**Intervention:** Every PwS was offered up to eight sessions of FES-UPP therapy, each lasting ~1 h, over a period of up to 6 weeks.

**Primary and secondary outcome measures:** The operation, acceptability, and feasibility of the interventions were assessed using video rating and the Wolf Motor Function Test Functional Ability Scale (WMF-FAS), direct observations of sessions and questionnaires for therapists and PwS.

**Results:** The system enabled 24% (Rater A) and 28% (Rater B) of PwS to carry out a wider range of functional tasks and improved the way in which the tasks were performed (mean scores of 2.6 and 2.2 (with FES) vs. mean scores 1.5 and 1.3 (without FES) (*p* < 0.05).

**Conclusion:** The FES-UP proved feasible to use in three different clinical environments, with PwS who varied widely in their impairment levels and time since stroke. Therapists and therapy assistants from a wide range of backgrounds, with varying degrees of computer and/or FES knowledge, were able to use the system without on-site technical support.

## Introduction

It is estimated that each year more than 100,000 stroke cases occur in the UK [The (1)]. Although the incidence of stroke has been slowly declining, acute treatments and hence survival rates have been improving significantly and the numbers living with stroke in the UK are increasing (2). Of those who survive a stroke only between 40 and 70% regain dexterity, depending on the initial severity of stroke (3). The ability to reach, grasp and manipulate objects is essential for independent accomplishment of daily tasks (4) and regaining upper limb function is a priority for people with stroke (PwS). Upper limb deficits have a direct effect on the ability to participate in functional and social aspects of life, which in turn influences quality of life (5).

A number of studies (6–8) have shown that high intensity, activity specific practice, supported by appropriate technology, can have a significant positive impact on upper limb motor recovery following stroke. However, although current clinical practice in treating people early after stroke varies between centers and across countries, it is generally characterized by low, or very low doses of activity-specific practice (9, 10) and limited use of suitable technology (11). Technology-based interventions not only offer the possibility of delivering high doses of functional activity practice without equivalent staffing demands, they also offer certain functionality not possible with traditional manual therapy approaches (12). Of key relevance to this study, unlike traditional therapy approaches, functional electrical stimulation (FES) directly activates lower motor neurons and associated sensory systems, which in turn has been shown to have effects on cortical activity (13). Further, appropriately delivered FES uniquely offers the opportunity for activity-specific practice using only the subject's own (impaired) neuromuscular system, rather than with assistance from a therapist, or a robotic device.

A number of reviews have suggested that FES may have a beneficial effect on upper limb motor recovery, if delivered in an appropriate way to the right patients, particularly early after stroke (14, 15). Electrical stimulation also has the potential to offset muscle decline associated with aging (16). Evidence from a recent study suggested that the therapeutic effect seen following a period of FES use is seen only in patients who retain the ability to produce brain activation patterns associated with movement planning (17) suggesting that an association of FES-induced movement with planned movement may be key to FES-supported recovery. Many previous devices have greatly limited the opportunity for the voluntary engagement with functional tasks which evidence suggests may be pivotal to recovery (17, 18). However, the devices used in many of the upper limb FES studies included in recent reviews typically stimulate only a small number of muscles, and offered little or no flexibility in how the FES was controlled.

In light of the promise offered by FES and the limitations with current systems we have developed a new FES system which will support patients to practice a range of voluntary controlled, FES-assisted upper limb functional activities. The new system, FES-UPP, described in detail in Sun et al. (19), allows therapists to set up sequenced patterns of electrical stimulation, bespoke to the particular activity being practiced, and patient's pattern of upper limb impairment.

This paper reports on a three center clinical investigation designed with the primary aim of demonstrating compliance of the new FES system with relevant essential requirements of the EU Medical Device Directive (20). Specifically, the primary aims of the study were to:

1. “Verify that, under normal conditions of use, the performance characteristics of the device are those intended by the manufacturer” (Essential Requirement 3). Normal conditions of use referred to therapist supervised use in a range of different clinical settings. For this study we used the term “therapist” to cover the range of professionals trained and deployed in the UK NHS to support, in a variety of ways, upper limb recovery in stroke.
  Performance characteristics intended by the manufacturer in this investigation were that use of the FES-UPP enabled participants to perform a wider range of FES supported functional activities, and/or perform the same activities in an improved way (19).
2. Determine any undesirable side effects and assess whether these constitute risks when weighed against the intended performance of the device (Essential Requirement 6).
  Secondary aims were to gather data to inform future efficacy studies (recruitment rates, and reasons for (non)-recruitment, patient characteristics, and the feasibility of the outcome measures) and to evaluate the system's usability (observed and reported errors and other usability issues from both the patient and therapist perspectives, together with setup times).

## Methods

### Study Participants

We aimed to recruit nine therapists and thirty PwS across the three sites. Site X: An in-patient stroke unit; Site Y: A combined Early Supported Discharge (ESD) and community service; Site Z: An outpatient clinic and in-patient stroke unit. All participants provided informed written consent to participate in the study.

Therapists were recruited if they worked with stroke patients and had successfully completed the training to use FES-UPP. In order to test the range of patient participant presentations the new FES-UPP device could be used with, we sought to recruit a heterogeneous group. Eligibility criteria for people with stroke were as follows:

#### Inclusion Criteria

Aged 16 or over; evidence of a clinical stroke; impairment of one or both upper limbs, for which they are, or plan to be, participating in therapy; medically fit to engage in active therapy sessions; sufficient level of cognition and communication to comply with the assessments and participate in the study; participant expected to remain under the care of one of the services for sufficient time to allow at least two sessions with FES-UPP to be completed.

#### Exclusion Criteria

Any neurological condition that affects voluntary control of upper limb movements; complex regional pain syndrome; orthopedic conditions that restrict joint range; severe Rheumatoid Arthritis; epilepsy not adequately controlled by medication; cardiac pacemaker or other active implanted device; metal external fixator implant; cancerous tissue/malignancy in the region of stimulation; pregnancy; skin rash, allergy, broken skin, wound, or poor skin condition in an area where electrodes are to be placed; requiring an interpreter.

A number of criteria were flagged as needing to be discussed with the Research Team before recruitment occurred:

Painful shoulder, or pain in the upper limb; participating in another study; fixed flexion contracture or excessive spasticity in more than two muscles; any medical condition other than those listed above that may affect the response to ES.

### Overview of FES-UPP System

**Figure 1:**
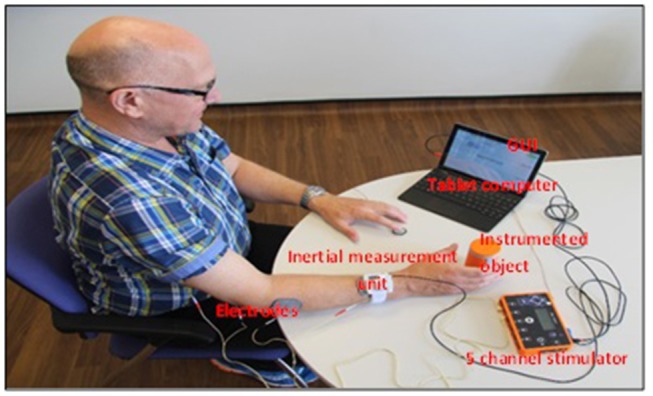
The FES-UPP system (the model in this figure has provided written informed consent to publish this photograph).

The FES-UPP system design is described in detail in Sun et al. (19) and shown in Figure 1.

In brief, the system consists of a dedicated tablet computer, a Graphical User Interface (GUI), using which the therapist sets up the FES controller, a five channel stimulator with electrodes and associated cables, up to four inertial measurement units with associated cables, an instrumented object which provides input to the controller, and a manual push button.

The tablet based setup software guides therapists through setting up the system. The setup process consists of five stages:

1. Selection and modification of activities from the FES-UPP upper limb activity library which contained 17 functional activities, and/or creation of new activities. The components of the FES-UPP activity library are provided in Table 1. Stage 1 guides the therapist in creating, modifying and selecting functional activities for the patient. Each activity is defined as a sequence of movement phases and their associated set of muscle(s) to be stimulated.
2. Donning of electrodes and sensors and set up of channels. In stage 2, the therapist is required to associate stimulation channels with the muscles chosen in Stage 1, to check that the placement of each electrode produces the right response, to set maximum and motor threshold for each channel, and to decide on the set of sensors to be used. If body-worn inertial measurements are to be used, the therapist also assigns the sensor(s) to appropriate body segment(s).
3. Set up of stimulation parameters for each movement phase for each of the selected activities. In this stage of setup, the therapist is required to set up suitable stimulation profiles for each muscle in each phase for each of the selected activities. A stimulation profile consists of a delay (s), ramp time (s) and target intensity value (μs). The device frequency was set at 40 Hz. Once the therapist is satisfied that the stimulation profiles are acceptable, the whole activity can be attempted by the patient, with the therapist moving between movement phases by pushing a button. During each ES-assisted attempt at the activity that the therapist decides is satisfactory, data from any of the assigned sensors, together with time-since-entering the phase is logged by the software, for use in stage 4.
4. Set up of transition rules. Once the therapist is satisfied with the stimulation profiles for each muscle in each phase, stage 4 involves defining the transition rules for progressing from one phase to the next. A transition between two successive phases can be triggered by a button press, a timeout, a change in body segment angle since entering the phase, instrumented object functions, or a logical combination of two of these events. Data collected during stage 3 is used to guide the therapist in this process.
5. Set up of patient instructions and biofeedback. In this stage the therapist sets up patient and activity-specific instructions and feedback on performance during practice of the activities.

**Table 1:** Components of the FES-UPP activity library.

**Activity category**	**Name of activity**
**A. Simple movements**	• Pendular shoulder exercises (with scapula stabilization)
**B. Unilateral activities**	
i. With scapula stabilization	• Reaching forwards to target • Sweeping coins • Wash face with sponge • Dusting away from body
ii. Without scapula stabilization	• Sweeping coins • Dusting across body • Wash unaffected arm • Picking up mobile phone • Drink from beaker • Eat using spoon • Place object on shelf
**C. Asynchronous bimanual activities**	• Hold and open jar • Pour from bottle into glass • Cut up food
**D. Synchronous bimanual activities**	
i) With scapula stabilization	• Sit to stand using arm support
ii) Without scapula stabilization	• Place tray on shelf

After completing the five setup stages, the therapist can leave this part of the software and enter the “Session Manager,” which allows the PwS to practice the chosen functional activity(s), and provides feedback to the therapist and PwS on their performance, both during and after practice.

### Study Design

The study design was based on a body of evidence supporting the value of intensive [(21), task-specific, electrical stimulation assisted (ES) (15)], voluntary initiated (17) practice in either speeding up, or improving recovery of upper limb function (22). The focus of our study was to demonstrate that the FES-UPP system could be used in clinical settings to deliver task-specific, high intensity, voluntary initiated, ES-assisted task practice.

### Patient Recruitment

All potential participants were provided with a Patient Participant Information Sheet and Patient Participant Invitation Letter explaining the purpose and content of the study. In cases where the potential participant was judged to have communication difficulties, the recruiting team used an alternative, adapted Patient Participant Information Sheet and consent form, developed with the help of a specialist Aphasia group. Potential participants were given as much time as they require to consider the information, with a minimum of 24 h. All participants provided informed written consent to participate in the study.

### FES-UPP Sessions

Each center was provided with three FES-UPP systems. A clinician manual and on-line training resource accompanied the system to supplement the clinician training, and provide instructions on using the FES-UPP.

Every PwS was offered up to eight sessions of therapy using the FES-UPP system, each lasting ~1 h, over a period of up to 6 weeks. The frequency and duration of therapy sessions was informed by the project advisory group, which included therapists and PwS. In order to conform to standard care there was no set schedule for when sessions should happen. Instead therapists were encouraged to use the system with each patient, as appropriate, for up to eight sessions. The operation, acceptability, and feasibility of the interventions were assessed using video rating as the primary outcome measure, direct observations of sessions and questionnaires for therapists and PwS.

**Figure 2:**
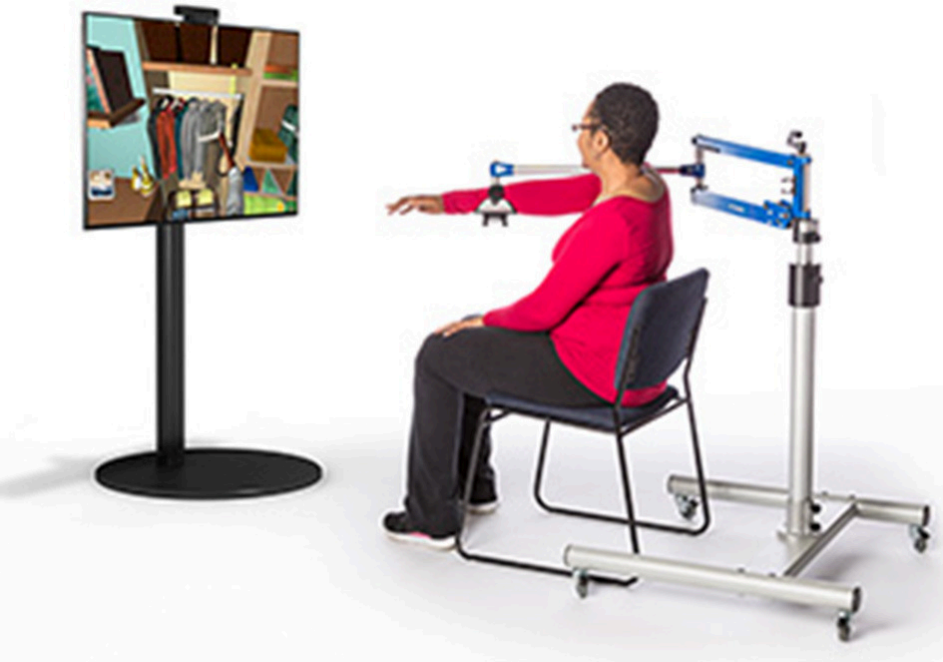
Saebo MAS de-weighting system. Clinical media reproduced with permission from SaeboUK Ltd and consent to publish https://www.saebo.com/media/.

The therapist and PwS agreed on one or more activities to be practiced using the FES-UPP system. Activities were selected based on clinical assessment and were such that they were difficult, or impossible for the PwS to perform unaided. The therapist set up the sequenced patterns of stimulation to multiple muscles to support the PwS in performing each of the functional activities, and where appropriate so that the system provided appropriate feedback and/or instruction. The PwS was encouraged to practice, under supervision, each of the FES-supported activities, until they indicated a desire to stop, or the session outcomes had been achieved. Center X piloted the use of the FES-UPP system in combination with an upper limb de-weighting system (Figure 2), for PwS who required additional assistance to move their arm against gravity.

### Capture of the Primary Outcome Data

**Figure 3:**
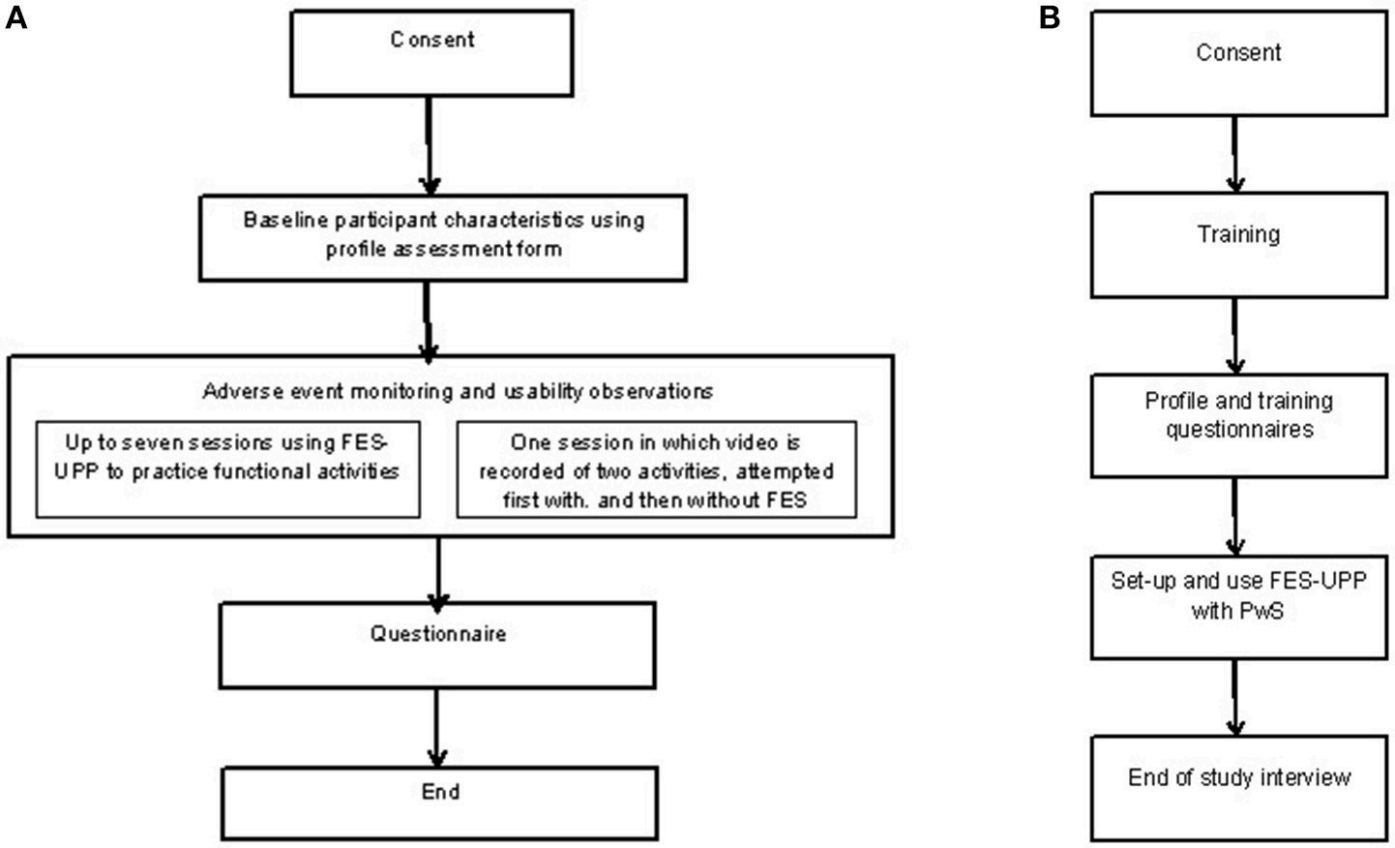
**(A,B)** Flow diagrams of the data collection protocols for both the PwS and therapists.

The primary outcome data was collected in a separate session to the standard FES-UPP treatment sessions (Figure 3A). The purpose was to address the primary research aim of verifying the performance characteristics, specifically that use of the FES-UPP enables PwS to perform a wider range of functional activities; and/or perform the same activities in an improved way. During one of the sessions the researcher video recorded each attempt at each activity for later analysis. Where possible, this session took place in the second or third treatment session to avoid the risk of discharge before the outcome measure could be taken. Session 1 was avoided to allow all participants to familiarize themselves with the system. In addition to the PwS and the treating therapist, a member of the research team was also present. The researcher, therapist and PwS agreed on up to two suitable unilateral activities, one or both of which may have been used in a previous session. As this type of session was to evaluate the performance of the system, and in order to reduce the effects of inter-therapist skill variability in using the system, the study researchers advised where necessary on the optimum setup of the FES-UPP system. The setup was fine-tuned to allow a robust attempt at the activity suitable for recording. The software was used to set all stimulation levels in each phase to zero and the PwS was asked to attempt the activity unaided (without FES). The FES stimulation levels were then re-instated the PwS was asked to attempt the activity with FES support. The process was repeated for a second activity where possible.

Members of the local research team were responsible for identifying adverse or serious adverse events. The local investigator was responsible for documenting the event and contacting the Principle Investigator to decide on the most appropriate course of action.

### Enrolment, Follow-Up, and Analysis

**Figure 4:**
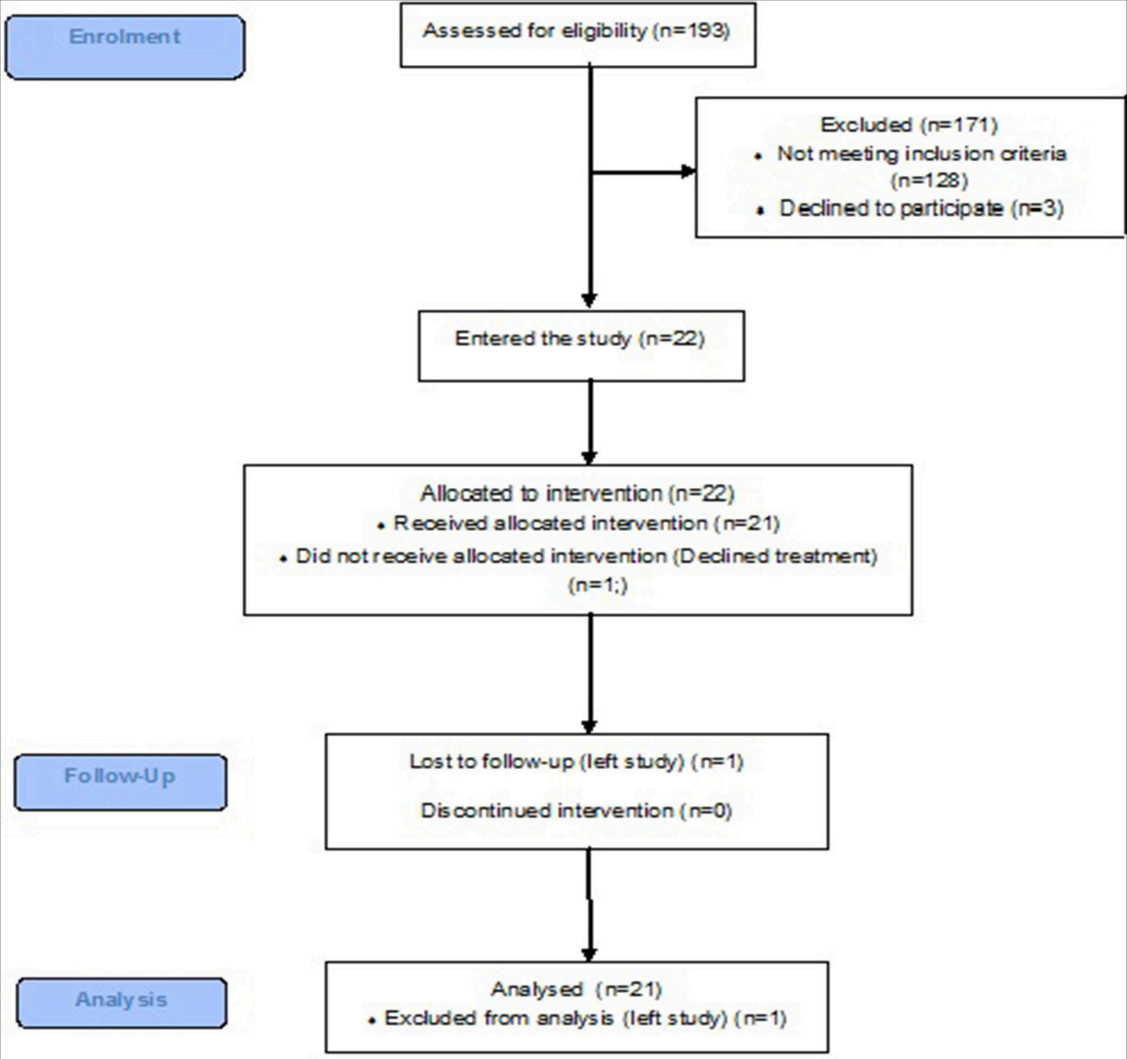
A CONSORT flow diagram showing patient enrolment, follow-up, and analysis at Centers X and Y.

The local research team at each site screened PwS for eligibility to enter the study. A full set of enrolment data was only available from Centers X and Y. A summary of the data for enrolment, follow-up and analysis is provided in the Consort flow diagram in the results section (Figure 4).

### Study Outcome Measures and Analysis

The study outcome measures are summarized below and categorized in accordance with the aims of the study.

#### Primary Aim 1: Demonstration of Compliance With Relevant ER of EU Medical Devices Directive

(a) To assess the primary outcome of the study i.e., to evaluate whether use of the FES-UPP enables PwS to perform a wider range of functional activities, and/or perform the same activities in an improved way, video recordings from FES-UPP outcome data collection sessions were taken of up to 2 functional tasks (1 or 2 tasks whichever was feasible for the PwS). An adapted version of the scoring system used in the Wolf Motor Function Test Functional Ability Scale (WMF-FAS) (23) was used (Table 2). Two rater's blinded to the condition (i.e., with, or without FES), evaluated randomized video footage of each activity using the six point ordinal WMF-FAS on two separate occasions. Paired blinded observations of performance on up to two upper limb activities (with FES-UPP compared with the patient's unaffected arm vs. without FES-UPP, again compared with their unaffected arm) were made for each PwS. In order to identify consistency of rating across the two rater's, inter-rater, and intra-rater reliability analysis was carried out. In order to determine the consistency of ratings, the two rater's conducted two separate ratings of the video footage within 1 week.

**Table 2:** An adapted version of the Wolf Motor Function Test Functional Ability Scale (WMF-FAS).

0 = Does not attempt with upper extremity (UE) being tested (no movement in spite of attempt)
1 = UE being tested does not participate functionally; however, an attempt is made to use the UE (Guidance notes: some movement present, not functional)
2 = Does, but requires more than two attempts to complete, or accomplishes very slowly (Guidance notes: accomplishing task, very slowly, gross effort)
3 = Does, but movement is influenced to some degree by synergy or is performed slowly or with effort (Guidance notes: slow, moderate effortful with synergy)
4 = Does; movement is close to normal^*^ but slightly slower; may lack precision, fine coordination, or fluidity (Guidance notes: minimal effort)
5 = Does; movement appears to be normal^*^

The guidance notes are intended to provide additional instructions to rater's to aid clarity.

* *For the determination of normal, the less-involved UE can be utilized as an available index for comparison, with premorbid UE dominance taken into consideration*.

To evaluate whether use of FES-UPP enables participants to perform activities in an improved way, paired observations of performance on up to two upper limb activities (with vs. without FES-UPP) were made for each PwS. A Wilcoxon Signed-Ranks test was used to test for differences in performance with vs. without FES-UPP (*p* < 0.05).

To test whether FES-UPP enabled participants to perform a wider range of activities, each of the WMF-FAS scores were reduced to categorical data. If an attempt scored a 0 or 1, the attempt was deemed to be a failure. If an attempt scored 2–6, the attempt was deemed a success. The McNemar test was used to test for differences in number of activities achieved with vs. without FES-UPP (*p* < 0.05).

#### Primary Aim 2: To Determine Any Undesirable Side Effects and Assess Whether These Constitute Risks When Weighed Against the Intended Performance of the Device

A record of adverse events was documented using a standard study designed proforma.

#### Secondary Aims

1) To gather data to inform future efficacy studies

Recruitment rates and reasons for non-recruitment were recorded at each site. Descriptive statistics were used to summarize the key findings.

To record the characteristics of PwS, a profile assessment form was completed within the first week (Table 2) to record date of birth, contact details, GP, date of stroke, side affected, dominant hand, spasticity measured using the Modified Ashworth Scale (24), upper limb impairments measured using the Fugl-Meyer upper extremity scale (25), neglect measured using the Star Cancellation Test (26), and cognitive ability measured using the Montreal Cognitive Assessment (27).

In order to characterize the therapist participant's their professional role, grade, level of experience in clinical practice, pre-study experience with FES and amount & type of computer use were recorded using the therapist profiling questionnaire.

The FES-UPP software logged the information during each therapy session onto the tablet in real time (28). The information logged by the software summarized the patient setup information, events that occurred in the FES-UPP software and information corresponding to each repetition of activity. The patient setup information contained the core setup information, including Finite State Machine (FSM) parameters (e.g., number of phases, phase name(s), muscles to be stimulated during each phase), stimulation parameters that defined stimulation profiles (pulse width, ramp time, delay time, and motor threshold), and transition rules etc. Events that occurred in the FES-UPP software were timestamped and logged when the user logged into and out of the software, entered different setup stages or when practicing within the session manager. Information corresponding to each repetition of activity included the number of repetitions, time spent in each movement phase, reason(s) for leaving each movement phase, and whether a successful repetition was achieved. A successful repetition was deemed to have occurred when the FSM had progressed through each movement phase and returned to the neutral phase.

The amount and type of therapy delivered, number of sessions, number of repetitions, activity type, practice time, and setup time per participant was recorded. Setup time was defined as the time taken for the therapist to progress through stages 1 to 5 of the setup process. Setup time was compared with practice time and degree of upper limb impairment. Type of activities chosen for each of the three sites and has been reported in a previous paper (19).

2) Usability

To evaluate the usability of the FES-UPP from the therapist's perspective, therapists were asked to complete a short form at the end of each session. Members of the research team also observed sessions where they recorded the details of any usability issues on a study designed usability form (Figure 3A).

To evaluate the usability of the FES-UPP from the PwS perspective we asked them to complete a short questionnaire (aided by the therapist) at the end of their time in the study (See Figure 3B and Supplementary Figure 1). Four respondents had communication difficulties, resulting in an adapted version of the questionnaire being used e.g., a numeric Likert scale and pictures to assist with answering the questions. This was developed with input from a specialist aphasia group.

### Data Analysis

Participant characteristics (PwS and therapists) and frequency of use of the FES-UPP system by therapists were analyzed using descriptive statistics.

The videos of participants attempting activities with and without FES were analyzed as follows. First, the quality of each video was assessed one of the study researchers determine if it was suitable for subsequent analysis. For the set of videos identified, inter-rater agreement on the quality of upper limb movement (WMF-FAS) was assessed using a weighted Kappa. In order to determine the consistency between rater's in assessing the quality of movement for the upper limb during a functional task, the WMF-FAS scores for all patients (with and without FES combined) were compared between rater's using a weighted Kappa (κ_w_) with linear weights (29). This was calculated twice, because each rater scored the videos on two occasions.

Kappa values were interpreted as follows: below 0.0 poor, 0.0–0.20 slight, 0.21–0.40 fair, 0.41–0.60 moderate, 0.61–0.80 substantial, 0.81–1.00 almost perfect (30).

To test whether FES-UPP allowed PwS to perform the same activities in an improved way, a Wilcoxon signed-rank test was used to test for differences in WMF-FAS with or without the FES-UPP.

To test whether the FES-UPP system enabled participants to perform a wider range of activities, each of the WMF-FAS scores were reduced to categorical data. If an attempt scored a 0 or 1, the attempt was deemed to be a failure. If an attempt scored 2–6, the attempt was deemed a success. An exact McNemar's test was used to test for differences in number of activities achieved with vs. without FES-UPP (*p* < 0.05).

The quantitative questionnaire data (Likert scale) were analyzed using descriptive summary statistics. The open-ended questions provided qualitative data that were grouped into themes.

Usability feedback from the therapist collected from observations of them using the system was analyzed using descriptive statistics to identify the key usability issues in each stage of the setup process. Setup time data was collected using the FES-UPP therapy session data logging system (28). Mean setup times were calculated for each center. The relationship between mean setup times and upper limb impairment was analyzed using correlation methods. All statistical analysis was completed using SPSS v24 (*p* < 0.05).

## Results

### Recruitment

Only screening data from Centers X and Y were available. Across these centers, 193 PwS were screened of which 171 were excluded. One hundred and twenty eight were ineligible, reasons being not a CVA (TIA or dementia) (*n* = 21), no, or resolved upper limb weakness (*n* = 31), severe cognitive impairment (*n* = 25) discharged from hospital (*n* = 22), medically unfit (*n* = 8), other conditions (*n* = 6), required an interpreter (*n* = 5), more than 6 months post stroke (*n* = 4), fixed contractures/spasticity (*n* = 4), uncontrolled epilepsy (*n* = 2) Very few patients declined to participate (*n* = 3), and 40 did not take part for other reasons (Figure 4).

Patients who met the inclusion criteria (*n* = 22) were consented into the study. One patient declined treatment after giving consent due to personal circumstances and was therefore withdrawn from the study, and any subsequent data analysis.

### Study Participant Characteristics

Table 3 summarizes the characteristics for the 22 PwS who were recruited into the study. We successfully implemented the study in three centers with different stroke populations. As the data for Center Z was taken from an in-patient stroke unit and an out-patient clinic, the data was categorized into acute/sub-acute (< 6 months) and chronic (>6 months). The median age of PwS was 67 (IQR 55.5–78.75). Age was similar across Centers X, Y and the in-patient facility at Center Z, and older at the out-patient clinic, with a median age of 73 (IQR 54.75–77.75). There were almost twice as many male participants than female, 71% were right hand dominant. As expected the participants at Site X (the acute/sub-acute stroke center) were very early post-stroke (range 1–3 weeks). Center Y (ESD & community) predominantly recruited from the community service rather than the ESD service, resulting in a median time since stroke of 22 weeks. The median length of time since stroke among outpatients at Site Z was much higher, 134 weeks. Side of stroke was similar across all centers. Upper limb impairment, measured using the Fugl-Myer upper extremity scale, had a median level of 8, indicating severe impairment, and was lowest (median 4) in the acute/sub-acute stroke ward, Center Z.

**Table 3:** Descriptive statistics for PwS study participants (median, IQR for age in years, gender, hand dominance, time since CVA (weeks), side of stroke and Fugl-Myer UE score) (FMA-UE).

**Description**	**Center X** **(*n* = 7)**	**Center Y** **(*n* = 4)**	**Center Z**		**Totals across all centers** **(*n* = 21)**
			**Acute/sub-acute** **(*n* = 5)**	**Chronic** **(*n* = 6)**	
Age (years)	Mean 63 (±20.94)	Mean 63 (±20.94)	Mean 65.2 (±14.98)	Mean 66.66 (±15.62)	Mean 65.47 (±14.58)
	Median 60	Median 67	Median 61	Median 73	Median 67
	IQR 52.5-81.5	IQR 65.75-68.5	IQR 60-78	IQR 54.75-77.75	IQR 55.5-78.75
Gender	6M (86%)	2M (50%)	1M (25%)	5M (83%)	14M (64%)
	1F (14%)	2F (50%)	4F (75%)	1F (17%)	8F (36%)
Hand dominance	5R (71%)	4R (100%)	5R (100%)	6R (100%)	20R (91%)
	2L (29%)				2L (9%)
Affected side	4R (57%)	1R (25%)	3R (60%)	2R (33%)	10R (45%)
	3L (43%)	3L (75%)	2L (40%)	4L (67%)	12L (55%)
FMA-UE/66	Median 8	Median 10.5	Median 4	Median 10	Median 8
	IQR 5-11	IQR 7.25-18.25	IQR 4-4	IQR 7.75-10.75	IQR 4.25-11.75
Time since CVA (weeks)	Median 0.78	Median 22	Median 6	Median 134	Median 7
	IQR 1-1.5	IQR 17.5-23.75	IQR 3-7	IQR 79-252	IQR 2-47

Eleven therapists were recruited across the three centers, comprising three Physiotherapists, two Occupational therapists, one Nurse, three Therapy Assistants, and two Clinical Engineers. Their mean clinical experience of treating PwS was 11.27 years. Four of the treating therapists had previous experience of using FES (Table 4).

**Table 4:** Professional role, grade, level of experience in clinical practice, pre-study experience with FES and amount & type of computer use.

**Therapist ID**	**Role & grade**	**Clinical experience**		**Previous FES experience**				**Computer use**	
		**Patients** **treated**	**Length (years)**	**Use of FES**	**Type of FES**	**UL/LL**	**Frequency**	**Frequency**	**Type**
XOT1	OT Band 7	Stroke	15	N	-	-	-	D	C/SD/PR/BW
XPT1	PT Static Band 6	Stroke	6	Y	MD	LL	OC	D	C/SD/PR/BW
XRA1	Therapy assistant	Stroke	1	N	-			D	C/PR/WP
YOT1	OT Band 6	Comm Stroke/ESD	7	N	-	-	-	M	WP
YPT1	PT Band 7	Comm Stroke/ESD	16	N	-	-	-	D	C/SD/PR/BW
YPI1	Stroke Specialist Nurse/Coordinator	Stroke	12	N	-	-	-	M	C
YRA1	Assistant Practitioner	Stroke	10	N	-	-	-	D	C/SD/BW/PR
YRA2	Technical Instructor	Stroke	1	N	-	-	-	D	C/SD/BW/PR/WP
ZPT1	Lead Physio/Clinical Tutor	Stroke/MS/ABI/SCI	21	Y	Exercise stimulator Odstock Pace	UL/LL	D	D	C/BW/SD
ZCE l	Clinical Engineer	Stroke/MS/TBI/SCI	5	Y	Exercise stimulatorODFS Pace	LL	M	D	C/SD/BW
ZCE2	Clinical Engineer	Stroke/MS/TBI/SCI	30	Y	Exercise stimulator, Odstock Pace	UL/LL	D	D	C/SD/BW/PR/WP
			**Mean = 11.27 (±8.83)**						

X, Y & Z in the therapist ID depicts the center. Key to abbreviations provided below.

*BW, browsing web; C, communication; D, daily; F, fortnightly; M, monthly; MD, missing data; N, no; N/A, not applicable; O, once only; OC, occasionally; OT, Occupational therapist; PG, playing games; PR, patient records; PT, Physiotherapist; RA, Rehabilitation Assistant; S, spread sheets; SI, social interaction; SD, searching databases; W, weekly; WP, word processing, Y, yes*.

A total number of 123 FES-UPP sessions were administered across the three centers (Table 5). Center Z carried out the most FES-UPP sessions (*n* = 3 therapists, 65 sessions), followed by Center X (*n* = 3 therapists, 43 sessions) with Center Y (*n* = 5 therapists, 15 sessions) using the FES-UPP system the least. At Center Y the physiotherapist and nurse did not use the system. The highest ranked frequency of setup for the FES-UPP was the Therapy Assistant at Center X (XTA1 = 39), the clinical engineers at Center Z (*n* = 27 and 25), followed by the Occupational Therapist (*n* = 14), and Therapy Assistant 1 (YTA1 = 6) at Center Y.

**Table 5:** Total number of FES-UPP sessions per therapist, PwS across all study sites.

**Patient** **ID**	**Therapist ID**											**Total no. of sessions/patient**
	**XOT** **1**	**XPT** **1**	**XTA** **1**	**YOT** **1**	**YPT** **1**	**YPI** **1**	**YTA** **1**	**YTA** **2**	**ZPT** **1**	**ZCE** **1**	**ZCE 2**	
X01	3	5	5(5S)									5
X02	3	3(1S)	7(7S)									8
X03	1(1S)	5	7(6S)									7
X04	0	2	3(3S)									3
X05	4	1	8(5S)									8
X06	1	6(1S)	5(5S)									6
X07	2	2(2S)	4(4S)									6
Total no. sessions												43 (±1.77)
Y01				5(3S)	0	0	2(2S)	0				5
Y02				2(2S)	0	0	1(1S)	1				3
Y03				4(1S)	0	0	3(S)	0				4
Y04				3(3S)	0	0	0	0				3
Total no. sessions												15 (±0.95)
Z01									0	8(8S)	3	8
Z02									0	0	8(8S)	8
Z03									8(8S)	0	0	8
Z04									0	8(8S)	0	8
Z05									0	0	1(1S)	1
Z06									0	4(4S)	0	4
Z07									0	0	8(8S)	8
Z08									0	0	7(7S)	7
Z09									0	1(1S)	0	1
Z10									8(8S)	0	0	8
Z11									0	4(4S)	0	4
Total no. sessions												65 (± 2.87)
Total no. sessions / therapist	14 (1S)	24 (4S)	39 (37S)	14 (9S)	0 (0S)	0 (0S)	6 (6S)	1 (0S)	16 (16S)	25 (25S)	27 (24S)	Total no. sessions = 123 (± 25.05)

*CE, Clinical Engineer; OT, Occupational Therapist; PT, physiotherapist; PI, Nurse; SU, setup of FES-UPP*.

### Primary Outcome Measure Results

#### Demonstration of Compliance With Relevant ER of EU Medical Devices Directive

One hundred videos were identified as being of suitable quality to be taken forward for analysis for (25 without FES and 25 with FES (*n* = 50), for each of the two rater's (*n* = 100). The video footage was analyzed by the same rater's on two occasions = total of 200 videos rated).

There was substantial agreement between the two rater's on both occasions (30): the first round of rating results were κ_w_ = 0.665 (95% CI, 0.533–0.797), *p* < 0.05 and the second round κ_w_ = 0.667 (95% CI, 0.533–0.807), *p* < 0.05. As the second round of rating was marginally more consistent for the two rater's, it was used for the Wilcoxon signed-rank test statistical analysis.

In order to assess the primary outcome of the study, whether using FES-UPP enabled PwS to perform the same activities in an improved way, fifty video clips of PwS performing functional tasks with and without FES were rated by two rater's using the WMF-FAS. Rater A deemed that 17 (68%) of the video clips showed improved functional performance with FES-UPP *in situ*, 2 (8%) were deemed to not have improved, and 6 (24%) were unchanged. Rater B rated 16 video clips (64%) as showing improved functional performance with FES, 2 (8%) as showing no improvement, and 7 (28%) to be unchanged. For Rater A, the mean WMF-FAS score when using FES-UPP was 2.6, compared to 1.5 without FES-UPP, a difference of 1.1, and the difference was significant (*p* < 0.05). Rater B scored consistently lower both with and without FES-UPP, however the overall difference between conditions was similar: the mean score when using FES-UPP was 2.2, compared to 1.3 without FES-UPP, a difference of 0.9, and the difference was significant (*p* < 0.05) (Table 6).

**Table 6:** Descriptive primary outcome data and Wilcoxon-signed rank test result for Rater's A and B.

**Test**	**Rater A (*****n*** **= 25)**						**Rater B (*****n*** **= 25)**					
	**↑**	**=**	**↓**	**Mean WMF-FAS (with FES)**	**Mean WMF-FAS (without FES)**	***p*-value**	**↑**	**=**	**↓**	**Mean WMF-FAS (with FES)**	**Mean WMF-FAS (without FES)**	**Difference (%) *p*-value**
Wilcoxon-signed rank test	17 (68%)	6 (24%)	2 (8%)	2.6	1.5	*p* < 0.05	16 (64%)	7 (28%)	2 (8%)	2.2	1.3	*p* < 0.05

*↑, improved; = no difference; ↓, did not improve*.

#### Adverse Incidents

Two serious adverse events occurred during the study. These were reported to the authorities and sponsor and were deemed to be unrelated to the procedure or investigational device.

### Secondary Outcome Data

#### Usability Issues, Including Logged Usage Data, Observed Errors, Quantitative and Qualitative Feedback on the System, and Setup Time

The logged data showed that the patterns of stimulation used to support practice of the same activity varied significantly across participants. As an example, the set of stimulation targets used by different therapists to support participants to practice the “Drink from a beaker” activity are shown in Table 7. This finding supported the utility of our flexible system, in which the stimulation patterns can be selected based both on the patient's presentation and activity requirements.

**Table 7:** Stimulation targets used by therapists to support participants to practice the “Drink from a beaker” activity across movement phases.

**Participant ID & muscles stimulated**	**Stimulation values (μs) across movement phases**					
	**Starting position**	**Reach**	**Grasp**	**Lift**	**Replace**	**Release**
**Z03**						
Extensors	0	146	48	46	56	225
Flexors	0	0	224	176	196	0
Triceps	0	126	76	0	220	0
Biceps	0	0	0	120	0	0
**Z04**						
Anterior deltoid and triceps	0	122	112	0	132	132
Extensors	0	162	0	42	42	72
Flexors	0	0	182	42	42	0
Biceps	0	0	0	172	0	0
**Z06**						
Abductor pollicis longus	0	183	0	0	0	122
Extensors	0	0	0	0	0	12
Flexors	0	0	92	102	0	0
Triceps	0	42	42	0	62	0
Biceps	0	0	0	82	0	0

Trial researchers (*n* = 3) conducted observations of the set-up and practice of FES-UPP at all three centers, 15 observations in total. Each time a problem arose that prevented the therapist from progressing through the setup process and/or practice (Session Manager), this was defined as a usability error. Table 8 displays the frequency of usability errors in each stage. Most errors occurred at Stages 1, 2, and 3.

**Table 8:** Number of usability issues recorded in each stage of the setup process.

**Stage of setup**	**Frequency of usability errors**
Loading patient file	4
Stage 1: Selecting/editing activity	8
Stage 2: Donning electrodes & setting stimulation targets	11
Stage 3: Adjusting stimulation levels across all phases	11
Stage 4: Selecting transition rules	1
Stage 5: Instructions and feedback	3
Session manager	4
Total	42

The frequency of usability errors were analyzed to determine the main usability problems. In response to therapist, PwS and researcher feedback a number of changes were suggested for the FES-UPP system, for example: Stage 1: re-designed to make selection and modification of library activities more intuitive; stage 2: anatomical terms to be used to automatically populate the sensor information; stage 3: allow pulse width to be decreased without stimulation being on.

A total of 15 (68%) PwS completed a questionnaire. Given the low number of participants, we have combined strongly agree/agree and strongly disagree/disagree categories. Nine PwS (60%) enjoyed using FES-UPP, 8 (53%) found it quick to set up, none found it uncomfortable. All participants found it easy to work with the system. Five PwS (33%) did not use the sensors, however those that did found them comfortable to wear. Fourteen out of the fifteen participants (93%) would recommend the system to other PwS, with the majority (*n* = 13, 87%) stating they would continue using the system if it was available.

Of the 15 PwS who were able to provide qualitative feedback, eight PwS (53%) remarked on the positive experience of seeing and feeling their arm/hand move again and other health benefits such as greater relaxation of their limb, and a feeling of increased energy. Three respondents (20%) suggested that there should be more emphasis on the hand/fingers, giving more help with correctly placing the electrodes and for the activities to be of sufficient duration to promote recovery.

Seven respondents (47%) found the on-screen instructions easy to understand and use and one asked for more feedback/graphs on the screen. Two respondents (13%) only received instructions from their therapist rather than using the on-screen instructions. The majority of the centers did not make use of the feedback function, however of those that did one respondent (7%) wanted more feedback/graphs on screen. Six people (40%) asked for the system to be easier to setup so they could apply it independently. Suggestions were use of a glove, fewer wires and a larger screen, with a take home system seen as desirable.

**Figure 5:**
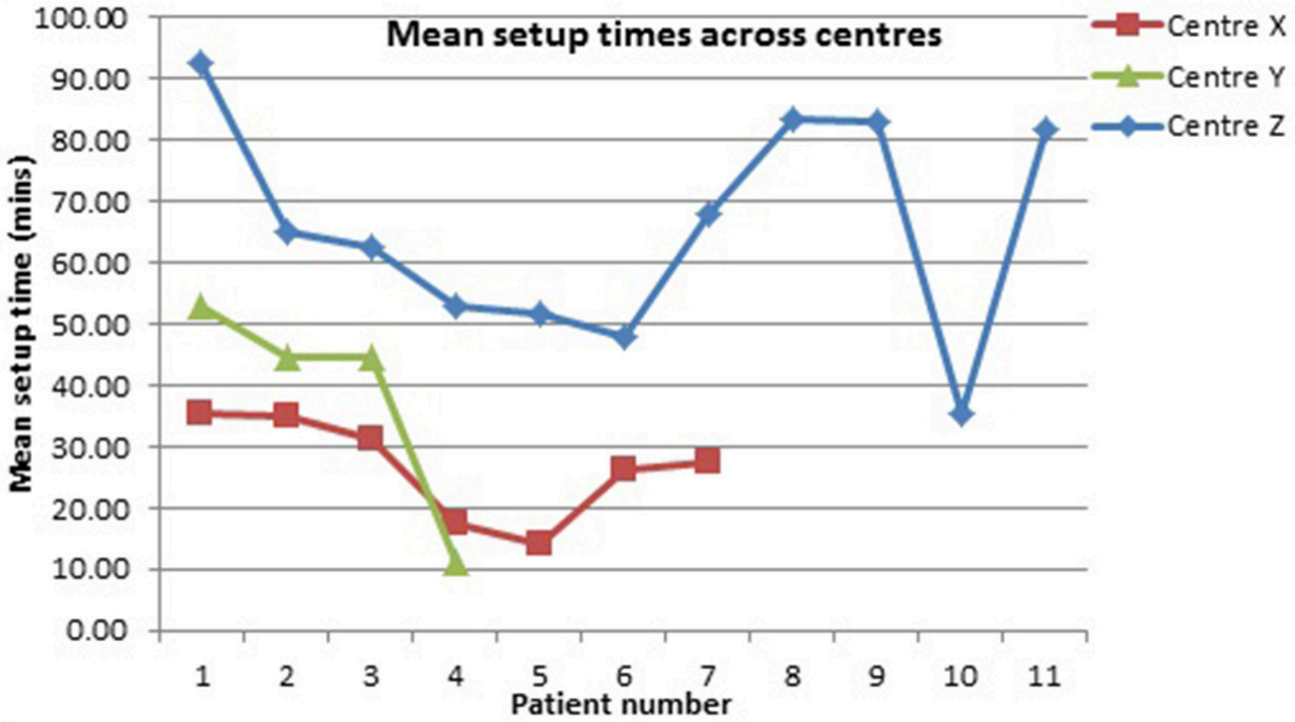
Mean setup times (mins) across Centers.

The setup time varied across centers (Figure 5). Our earlier (lab-based) work suggested setup time was positively correlated with impairment level (31).

There was no correlation between PwS level of impairment and length of time to setup the FES-UPP system. (*r* = −0.124, *n* = 21, *p* > 0.05).

## Discussion and Limitations

### Primary Aim of the Study

#### Demonstration of Compliance With Relevant ER of EU Medical Devices Directive

The primary aim of the study was to establish whether the FES-UPP system enabled participants to perform a wider range of activities and/or enabled participants to perform activities in an improved way. The system enabled 68% (Rater A) and 64% (Rater B) of PwS to carry out a wider range of functional tasks or improved the way in which the tasks were performed (mean WMF-FAS scores of 2.6 and 2.2 (with FES) vs. mean scores 1.5 and 1.3 (without FES) (Table 6). Of the video clips where rater's scored the stroke participants either the same or worse, the majority had a predominance of hypertonicity. This may suggest that a future efficacy study may consider restricting inclusion to those without significant spasticity.

The FES-UPP system allowed practice of upper limb functional activities within busy acute hospital (*n* = 12) and out-patients settings (*n* = 6), and in participants' homes (*n* = 4). Each setting presents its own challenges, short duration of stay for in-patient settings and logistical challenges such as finding a suitable surface to accommodate use of the FES-UPP, identification of functional objects for practice, and the cold temperature of some homes. The FES-UPP system proved to be sufficiently flexible to be used to support each participant. It successfully allowed stroke participants with a wide range of impairments to achieve active, functional practice that would not have been possible without FES.

No serious adverse events that were attributable to the FES-UPP system were noted demonstrating that the system was safe to use.

### Secondary Aims of the Study

#### PwS and Therapist Recruitment

The study was successful in recruiting and implementing FES in a varied stroke population, in terms of clinical settings and time since stroke, and with a range of different types of therapists. Although a high number of PwS were screened (*n* = 193), only 22 out of a target of 30 entered the study (Figure 4). Due to local differences each center screened in accordance with their own protocols which were not necessarily comparable. In future a standardized screening tool would ensure greater consistency of screening and subsequent recruitment. One of the reasons for exclusion from the study was an upper limb impairment that resolved early in the hospital stay. Due to new medical advances e.g., thrombolysis, a greater proportion of patients achieve some resolution of their impairments in the acute phase post stroke. The length of time that patients remain in hospital following a stroke has significantly decreased over the last decade (32), with the median length of stay estimated to be 7 days, resulting in some patient's being discharged before interventions can commence. In our study 11 PwS were deemed to be too high level in their upper limb recovery process to benefit from the FES-UPP system. Although people with acute stroke may have the potential to benefit more from FES-UPP than those at a later stage in their recovery (15) in common with other studies, including those studying less complex interventions (33), we found recruitment from this group to be difficult. For instance, at Center X, we screened 132 acute patients over 7 months and recruited 7 to the study.

Therapists were enthusiastic to take part in the study, with 11 from an original target of nine therapists recruited. However, one of the therapists YPI 02 sustained a broken wrist and was unable to take part in the remainder of the study. Staffing ratio's at Center Y were also affected by a staff bereavement. Therapist's previous experience of FES did not appear to influence their use of the FES-UPP system (Table 4).

### Usage and Usability of the FES-UPP System From the Therapist and PwS Perspectives

Patient participants' presentation, working practices, culture and personnel available to use the FES-UPP varied across centers, could have influenced how the system was used (Table 5). Center X utilized an inter-disciplinary approach where physiotherapist worked alongside occupational therapists and therapy assistants within the same treatment session. In this center, often the therapy assistant was nominated to setup the system whilst the physiotherapist and occupational therapist prescribed the activity to be practiced, and handled the limb where required. Generally the therapy assistants in this study used the system more frequently than qualified therapists. If a similar pattern of use was found in the future, this may both enhance the therapist assistants' role and have the potential to reduce treatment staffing costs.

Of the 17 activities in the FES-UPP activities library, sweeping coins and drinking from a beaker were the most frequently chosen activities. Therapists tended to opt to either modify an existing activity from the FES-UPP library, or create their own activity. Often the created activity was rather simple, for example reach to target and hand opening.

It is also worth noting that the study protocol somewhat constrained the therapists' use of what is a very flexible system. For instance, in the first few sessions, the therapists focused on identifying a suitable activity/activities with which to address the primary aim. It is likely that therapists would need to use the system for a more prolonged period and without the constraints of the protocol described here in order to fully exploit its potential. Future iterations of the system that increase the amount of guidance for the user, perhaps with a greater use of default settings, may help with setup and more effective use.

Direct observation of end users working with rehabilitation technologies presents many challenges, but the importance of “context of use” and usability testing in “real world” settings should not be underestimated. The usability observations in our study were an effective method of identifying issues that were problematic for therapists when using the system. Stages 2 and 3 of the setup process were the most complicated and therefore it was not surprising that it was in these stages that the highest number of usability issues was observed. Stroke participants were generally positive about the system with 14 out of 15 respondents reporting that they would recommend the system to other PwS, and 13 reporting they would have liked to have used the system for longer. Some PwS commented on the positive experience of seeing and feeling their arm/hand move again.

### Setup Time

The limited time available for rehabilitation, particularly the hemiplegic upper limb, is a frequently reported challenge in current UK health care settings. Our study concurred with these findings, with therapists reporting insufficient time to dedicate to the treatment of patients. This situation makes setup time for devices a critical factor in their adoption (10). Setup times in this study were variable and remained lengthy, with mean setup times (mins) of 26.7 at Center X, 38.32 at Center Y, and 65.89 at Center Z (Figure 5). The longer setup times at Center Z appeared to be influenced by the higher levels of hypertonicity common in patients with chronic stroke, suggesting future efficacy studies of FES-UPP should consider the issue of hypertonicity when defining inclusion and exclusion criteria. Further, two of the “therapists” at Center Z were bioengineers, and tended to set up more complex activities than most of the physiotherapists and occupational therapists involved in the study. Unlike findings from our previous work (31), setup time in this study appeared not to be related to level of impairment. However, our previous study was conducted under much more controlled conditions, in a laboratory setting with a single therapist, and undoubtedly other factors also influence the setup times seen in this study, conducted in the real clinical environment. It was observed that there were a number of sessions where therapist did not progress to the “Session Manager” stage, probably due to either running out of time, or being content to work with the system in (partial) setup mode; it is worth noting that from stage 3 of setup onwards the patient is practicing ES-assisted movements. An improvement in the level of automation of setup and consequent reduction in setup times, coupled with further training on the best approaches to using the system may help to alleviate this problem.

### Limitations

The study design proved very time consuming to implement and relied on the co-operation of therapists at three busy clinical sites across England. Recruitment proved difficult and a better screening tool is needed for future studies; the missing screening data from site Z suggests better training of clinical staff on the protocol was needed. The training offered to therapists prior to the study was adequate, but observations reported in our previous paper (19) suggesting that efficiency of setup may improve with time points, with more prolonged training being required in future studies. The primary outcome measure had not previously been used to compare performance of activities with FES to performance without FES and hence a minor modification was required. While it was shown to be adequate for its purpose with substantial agreement between the two rater's, it relies on inevitable subjectivity of rater's. Further, it proved time consuming to deliver and hence in some cases therapists were only able to record attempts at one, rather than two activities. Should a similar comparison be required in future studies, it would be useful to develop an objective metric based on the logged data, and possibly instrumented analyses e.g., kinematic and/or kinetic data.

## Conclusions

Despite the limitations discussed above, the study demonstrated that under normal conditions of use, the performance characteristics of the device were those intended by the manufacturer. Specifically, when using FES-UPP, patient participants were able to perform functional activities in an improved way and/or performed a wider range of functional activities than when attempting the same activities without FES support. The two serious adverse events observed in the investigation were unrelated to the intervention. The study also demonstrated the feasibility of using FES-UPP in three different clinical environments, with patient participants varying widely in their impairment levels (4–49 on the FM-UE scale) and time since stroke (1–936 weeks). Further, the results showed that therapists from a wide range of backgrounds, with varying degrees of computer and/or FES knowledge, were able to use the system without on-site technical support. Full patient screening data were gathered at two out of the three sites and recruitment data collected at all three sites. It was clear from these that the key barriers to patient recruitment were no or resolved upper limb weakness, a high level of cognitive impairment, or discharged from the service. Therapist recruitment was straightforward, although retention was a particular problem at one site, likely due to circumstances beyond our control. The outcome measures proved feasible to collect, as did the usability data. Direct observation of therapists/patients coupled with the data logged by the system, and post-study interview provided a depth of insight not usually reported in studies of this type of technology. Overall the system was viewed positively by PwS. Future studies should investigate whether the FES-UPP system's demonstrated capability to support patients to practice ES-supported functional activities leads to improvements in unassisted upper limb function.

## Ethics Statement

The study took place at three clinical centers and was approved by the NHS (ref: 16/YH/0258) and University of Salford Research Ethics Committees (HSCR 16-39). The applicants also received a notice of no objection from the UK Medicines and Healthcare Regulatory Authority to the use of the FES-UPP system for the purpose of the clinical investigation study (C1/2016/0034). The procedures employed in the study complied with ICH-GCP and The Declaration of Helsinki (34).

## Author Contributions

MS, DH, LK, and CS contributed to the conception and design of the study. MS and DH contributed conception and design of the FES-UPP FSM controller and setup software. HL, CS, PT, LK, and SC designed the clinical trials. CS wrote the first draft of the manuscript. MS wrote the setup software code. CS, HL, KW, PT, and EM supervised and collected the therapist and patient study data. PT, SF, and EM designed the hardware five channel stimulator. EM implemented the hardware five channel stimulator. EM, MS, and DH designed the communication protocol between FES-UPP software and hardware stimulator. All authors contributed to manuscript revision, read, and approved the submitted version. SC provided statistical advice. CS and MS equally contributed to preparation of this article.

### Conflict of Interest Statement

EM was employed by OML, PT was 40% seconded to OML from Salisbury NHS foundation Trust. PT holds shares in OML. The funder played no role in the study design, the collection, analysis or interpretation of data, the writing of this paper or the decision to submit it for publication. The remaining authors declare that the research was conducted in the absence of any commercial or financial relationships that could be construed as a potential conflict of interest.

## Supplementary Material

Figure S1 Usability questionnaire.

## References

[1] Stroke Association About Stroke (2015). Available online at: http://www.stroke.org.uk/about-stroke (Accessed Aug 22, 18).

[2] Lee S Shafe AC Cowie MR. UK stroke incidence, mortality and cardiovascular risk management 1999-2008: time-trend analysis from the General Practice Research Database. BMJ Open. (2011) 1:e000269. 10.1136/bmjopen-2011-000269 22021893 PMC3211058

[3] Houwink A Nijland RH Geurts AC Kwakkel G. Functional recovery of the paretic upper limb after stroke: who regains hand capacity? Arch Phys Med Rehabil. (2013) 94:839–44. 10.1016/j.apmr.2012.11.031 23201317

[4] Wolfe CDA. The impact of stroke. Br Med Bull. (2000) 56:275–86. 10.1258/0007142001903120 11092079

[5] Mayo NE Wood-Dauphinee S Côté R Durcan L Carlton J. Activity, participation, and quality of life 6 months poststroke. Arch Phys Med Rehabil. (2002) 83:1035–42. 10.1053/apmr.2002.33984 12161823

[6] McCabe J Monkiewicz M Holcomb J Pundik S Daly JJ Comparison of robotics FES, and motor learning methods for treatment of persistent upper extremity dysfunction after stroke: a randomized controlled trial. Arch Phys Med Rehabil. (2014) 96:981–90. 10.1016/j.apmr.2014.10.022 25461822

[7] Lo AC Guarino PD Richards LG Haselkorn JK Wittenberg GF Federman DG. Robot-assisted therapy for long-term upper-limb impairment after stroke. N Engl J Med. (2010) 362:1772–83. 10.1056/NEJMoa0911341 20400552 PMC5592692

[8] Wolf SL Winstein CJ Miller JP Taub E Uswatte G Morris D. Effect of constraint-induced movement therapy on upper extremity function 3 to 9 months after stroke: the EXCITE randomized clinical trial. JAMA. (2006) 296:2095–104. 10.1001/jama.296.17.2095 17077374

[9] Hayward KS Brauer SG. Dose of arm activity training during acute and subacute rehabilitation post stroke: a systematic review of the literature. Clin Rehabil. (2015) 29:1234–43. 10.1177/0269215514565395 25568073

[10] McHugh G Swain ID Jenkinson D. Treatment components for upper limb rehabilitation after stroke: a survey of UK national practice. Disabil Rehabili. (2013) 36:925–31. 10.3109/09638288.2013.824034. 23962194

[11] Hughes AM Burridge JH Demain SH Ellis-Hill C Meagher C Tedesco-Triccas L. Translation of evidence-based assistive technologies into stroke rehabilitation: users' perceptions of the barriers and opportunities. BMC Health Serv Res. (2014) 14:124. 10.1186/1472-6963-14-124 24620739 PMC4007558

[12] Thrasher TA Zivanovic V McIlroy W Popovic MR. Rehabilitation of reaching and grasping function in severe hemiplegic patients using functional electrical stimulation therapy. Neurorehabili Neural Repair. (2008) 22:706–14. 10.1177/1545968308317436 18971385

[13] Quandt F Hummel FC. The influence of functional electrical stimulation on hand motor recovery in stroke patients: a review. Exp Transl Stroke Med. (2014) 6:9. 10.1186/2040-7378-6-9 25276333 PMC4178310

[14] Farmer SE Durairaj V Swain I Pandyan AD. Assistive technologies: can they contribute to rehabilitation of the upper limb after stroke? Arch Phys Med Rehabil. (2014) 95:968–85. 10.1016/j.apmr.2013.12.020 24429002

[15] Eraifej J Clark W France B Desando S Moore D. Effectiveness of upper limb functional electrical stimulation after stroke for the improvement of activities of daily living and motor function: a systematic review and meta-analysis. Syst Rev. (2017) 6:40. 10.1186/s13643-017-0435-5 28245858 PMC5331643

[16] Kern H Barberi L Löfler S Sbardella S Burggraf S Fruhmann H. Electrical stimulation counteracts muscle decline in seniors. Front Aging Neurosci. (2014) 6:189. 10.3389/fnagi.2014.00189 25104935 PMC4109438

[17] Gandolla M Ward NS Molteni F Guanziroli E Ferrigno G Pedrocchi A. The neural correlates of long-term carryover following functional electrical stimulation for stroke. Neural Plasticity. (2016) 2016:4192718. 10.1155/2016/4192718 27073701 PMC4814690

[18] Maldonado MA Allred RP Felthauser EL Jones TA Motor skill training, but not voluntary exercise, improves skilled reaching after unilateral ischemic lesions of the sensorimotor cortex in rats. Neurorehabil Neural Repair. (2008) 22:250–61. 10.1177/1545968307308551 18073324 PMC2586983

[19] Sun M Smith C Howard D Kenney L Luckie H Waring K. FES-UPP: a flexible functional electrical stimulation system to support upper limb functional activity practice. Front Neurosci. (2018) 12:449. 10.3389/fnins.2018.00449 30026683 PMC6041417

[20] The Council of the European Communities Council Directive 93/42/EEC. 1993L0042 -EN−11.10.2007−005.001−2. Annexe 1. of 14 June 1993 concerning medical devices (1993).

[21] Jeffers MS Karthikeyan S Gomez-Smith M Gasinzigwa S Achenbach J Feiten A. Does stroke rehabilitation really matter? part B: an algorithm for prescribing an effective intensity of rehabilitation. Neurorehabil Neural Repair. (2018) 32:73–83. 10.1177/1545968317753074 29334831

[22] Lewthwaite R Winstein CJ Lane CJ Blanton S Wagenheim BR Nelsen MA. Accelerating stroke recovery: body structures and functions, activities, participation, and quality of life outcomes from a large rehabilitation trial. Neurorehabili Neural Repair. (2018) 32:150–65. 10.1177/1545968318760726 29554849 PMC5863583

[23] Wolf SL Thompson PA Morris DM Rose DK Winstein CJ Taub E. The EXCITE trial: attributes of the wolf motor function test in patients with subacute stroke. Neurorehabil Neural Repair. (2005) 19:194–205. 10.1177/1545968305276663 16093410

[24] Bohannon RW Smith MB. Interrater reliability of a modified Ashworth scale of muscle spasticity. Phys Ther. (1987) 67:206–7. 10.1093/ptj/67.2.206 3809245

[25] Fugl-Meyer AR Jääskö L Leyman I Olsson S Steglind S The post-stroke hemiplegic patient. 1. a method for evaluation of physical performance. Scand J Rehabil Med. (1975) 7:13–31. 1135616

[26] Halligan P Wilson B Cockburn J. A short screening test for visual neglect in stroke patients. Int Disabil Stud. (1990) 12:95–9. 10.3109/03790799009166260 2096121

[27] Nasreddine ZS Phillips NA Bédirian V Charbonneau S Whitehead V Collin I. The montreal cognitive assessment, MoCA: a brief screening tool for mild cognitive impairment. J Am Geriatr Soc. (2005) 53:695–9. 10.1111/j.1532-5415.2005.53221.x 15817019

[28] Sun M Joseph Kenney LP Howard D Smith C Waring K Luckie H Logging therapy session data via an upper limb FES rehabilitation system In: Annual Conference of the International Functional Electrical Stimulation Society. Nottwil (2018).

[29] Cicchetti DV Allison T A new procedure for assessing reliability of scoring EEG sleep recordings. Am J EEG Technol. (1971) 11:101–9. 10.1080/00029238.1971.11080840

[30] Landis JR Koch GG. The measurement of observer agreement for categorical data. Biometrics. (1977) 33:159–74. 10.2307/2529310 843571

[31] Smith C Joseph Kenney LP Howard D Waring K Sun M Luckie H Prediction of setup times for an advanced upper limb functional electrical stimulation system. J Rehabil Assis Technol Eng. (2018) 5:1–9. 10.1177/2055668318802561 PMC6531802 31191957

[32] Royal College of Physicians I.S.W.P., Sentinel Stroke National Audit Programme (SSNAP), in Clinical audit August-November, Public Report London (2017).

[33] Feldman WB Kim AS Chiong W Trends in recruitment rates for acute stroke trials, 1990–2014. Stroke. (2017) 48:799–801. 10.1161/STROKEAHA.116.014458 28104835 PMC5330837

[34] Declaration of Helsinki Recommendations guiding physicians in biomedical research involving human subjects. Br Med J. (1964) 313:1448.

