# Supplementary figures and images for: A Three-Site Clinical Feasibility Study of a Flexible Functional Electrical Stimulation System to Support Functional Task Practice for Upper Limb Recovery in People With Stroke
Source: Front Neurol. 2019 Mar 20;10:227. doi: 10.3389/fneur.2019.00227 (PMC6436422; doi:10.3389/fneur.2019.00227)

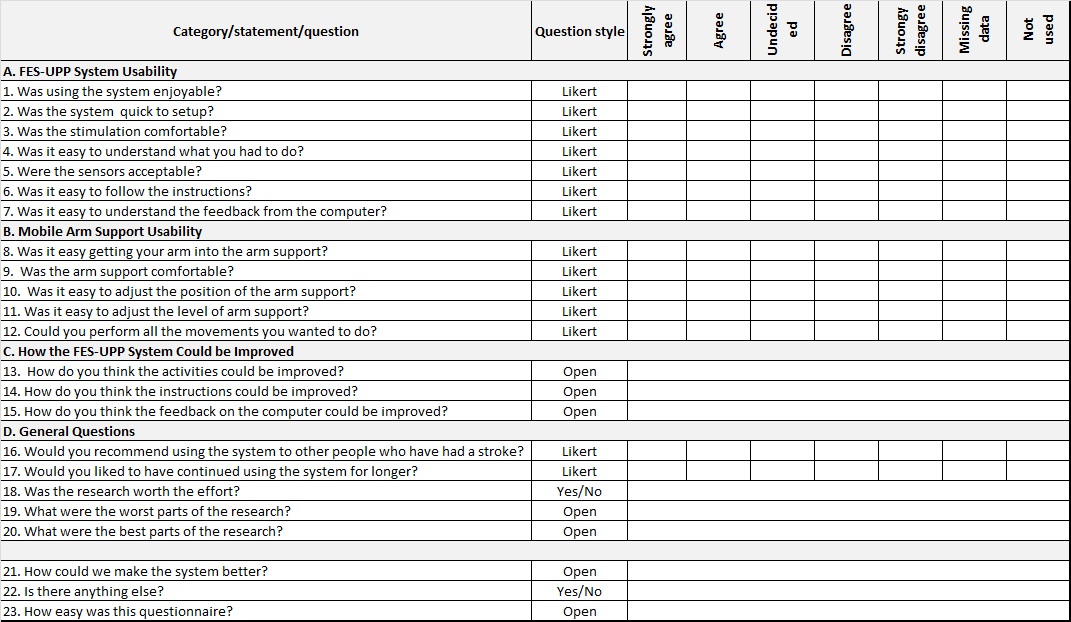

Supplement: Figure S1 — Usability questionnaire. [file Image_1.JPEG]
